# Diagnostic capabilities of artificial intelligence as an additional reader in a breast cancer screening program

**DOI:** 10.1007/s00330-024-10661-3

**Published:** 2024-02-22

**Authors:** Mustafa Ege Seker, Yilmaz Onat Koyluoglu, Ayse Nilufer Ozaydin, Sibel Ozkan Gurdal, Beyza Ozcinar, Neslihan Cabioglu, Vahit Ozmen, Erkin Aribal

**Affiliations:** 1 Department of Radiology, Acibadem Mehmet Ali Aydinlar University, School of Medicine, Istanbul, Turkey; 2 Marmara University, School of Medicine, Istanbul, Turkey; 3 Namik Kemal University, School of Medicine, Tekirdag, Turkey; 4 Istanbul University, School of Medicine, Istanbul, Turkey

**Keywords:** Mammography, Screening, Breast cancer, Artificial intelligence

## Abstract

**Objectives:**

We aimed to evaluate the early-detection capabilities of AI in a screening program over its duration, with a specific focus on the detection of interval cancers, the early detection of cancers with the assistance of AI from prior visits, and its impact on workload for various reading scenarios.

**Materials and methods:**

The study included 22,621 mammograms of 8825 women within a 10-year biennial two-reader screening program. The statistical analysis focused on 5136 mammograms from 4282 women due to data retrieval issues, among whom 105 were diagnosed with breast cancer. The AI software assigned scores from 1 to 100. Histopathology results determined the ground truth, and Youden’s index was used to establish a threshold. Tumor characteristics were analyzed with ANOVA and chi-squared test, and different workflow scenarios were evaluated using bootstrapping.

**Results:**

The AI software achieved an AUC of 89.6% (86.1–93.2%, 95% CI). The optimal threshold was 30.44, yielding 72.38% sensitivity and 92.86% specificity. Initially, AI identified 57 screening-detected cancers (83.82%), 15 interval cancers (51.72%), and 4 missed cancers (50%). AI as a second reader could have led to earlier diagnosis in 24 patients (average 29.92 ± 19.67 months earlier). No significant differences were found in cancer-characteristics groups. A hybrid triage workflow scenario showed a potential 69.5% reduction in workload and a 30.5% increase in accuracy.

**Conclusion:**

This AI system exhibits high sensitivity and specificity in screening mammograms, effectively identifying interval and missed cancers and identifying 23% of cancers earlier in prior mammograms. Adopting AI as a triage mechanism has the potential to reduce workload by nearly 70%.

**Clinical relevance statement:**

The study proposes a more efficient method for screening programs, both in terms of workload and accuracy.

**Key Points:**

*• Incorporating AI as a triage tool in screening workflow improves sensitivity (72.38%) and specificity (92.86%), enhancing detection rates for interval and missed cancers.*

*• AI-assisted triaging is effective in differentiating low and high-risk cases, reduces radiologist workload, and potentially enables broader screening coverage.*

*• AI has the potential to facilitate early diagnosis compared to human reading.*

## Introduction

Breast cancer is the most common cancer and the second leading cause of cancer-associated mortality in women [1]. Screening programs with mammography have contributed significantly to decreasing morbidity and mortality associated with breast cancer by enabling early diagnosis [2, 3]. Mammography shows high rates of false positives and false negatives that can be attributed to several factors including the following: dense breast tissue, interpretational errors, and incorrect positioning [4–6]. Artificial intelligence (AI) promises great potential in reducing errors and can improve specificity when used as a second reader in screening or as a computer-aided decision support system in decision-making [7–10]. This leads to a decreased workload for radiologists and positions AI as a potential triage element. A recent meta-analysis encompassing 15 individual studies focusing on both standalone detection (8 studies) and triage (7 studies) AI revealed that the efficacy of AI algorithms is approaching parity with human readers in tasks involving standalone computer-aided detection and computer-aided diagnosis [11]. Nevertheless, despite these encouraging outcomes, further AI studies are needed to establish clinically relevant thresholds in line with current reader performance and screening program objectives [11].

In mammography screening, recall and interval cancer rates are crucial benchmarks. Interval cancers carry the risk of being biologically more significant and larger, potentially impacting the effectiveness of mammography screening in reducing mortality rates [12, 13]. One contributing factor to these interval cancers is human errors in reading mammograms, influenced by factors such as fatigue, workload, and cognitive load [14]. AI holds significant potential in mitigating these errors and thereby reducing the incidence of interval cancers [8, 11].

On the other hand, despite the promising findings demonstrating the potential of AI in screening, there is a gap in the literature regarding the temporal associations between consecutive visits in a screening program approached longitudinally, with studies often assessing mammograms as separate studies instead of as part of a sequential analysis to detect missed and interval cancers [8, 9].

In this study, we aimed to evaluate the early-detection capabilities of AI in a screening program over its duration, with a specific focus on the detection of interval cancers, the early detection of cancers with the assistance of AI from prior visits, and its impact on workload for various reading scenarios.

## Materials and methods

### Population

The study was approved by the institutional review board (date, number: 15.10.2020, 2020–22/23). Digital mammography images were retrieved from the Bahcesehir Mammographic Screening Program (BMSP) [15]. All patients signed an informed consent form under BMSP. The Institutional Review Board has waived the need for informed consent for the retrospective evaluation of anonymized medical data for the current study. The BMSP study took place in the Bahcesehir district of Istanbul, Turkey, from January 2009 to January 2019 and included women aged 40 to 69 years. Out of the 8758 women invited to participate in the screening, there was an 85% participation rate over the course of five biennial rounds. The reporting of this study conforms to STROBE guidelines [16].

### Mammograms

Two views, mediolateral oblique (MLO) and craniocaudal (CC), were obtained with full-field digital mammography (Selenia, Hologic, United States of America). Two expert radiologists with more than five years of experience in breast radiology evaluated the images. In cases of discordance, a third radiologist with more than 20 years of breast imaging experience assessed the images for the final decision. The fourth edition of the Breast Imaging-Reporting and Data System of the American College of Radiology (BI-RADS) was followed [17]. The fifth edition of BI-RADS was not available at the beginning of the BMSP.

### Data retrieval

**Fig. 1:**
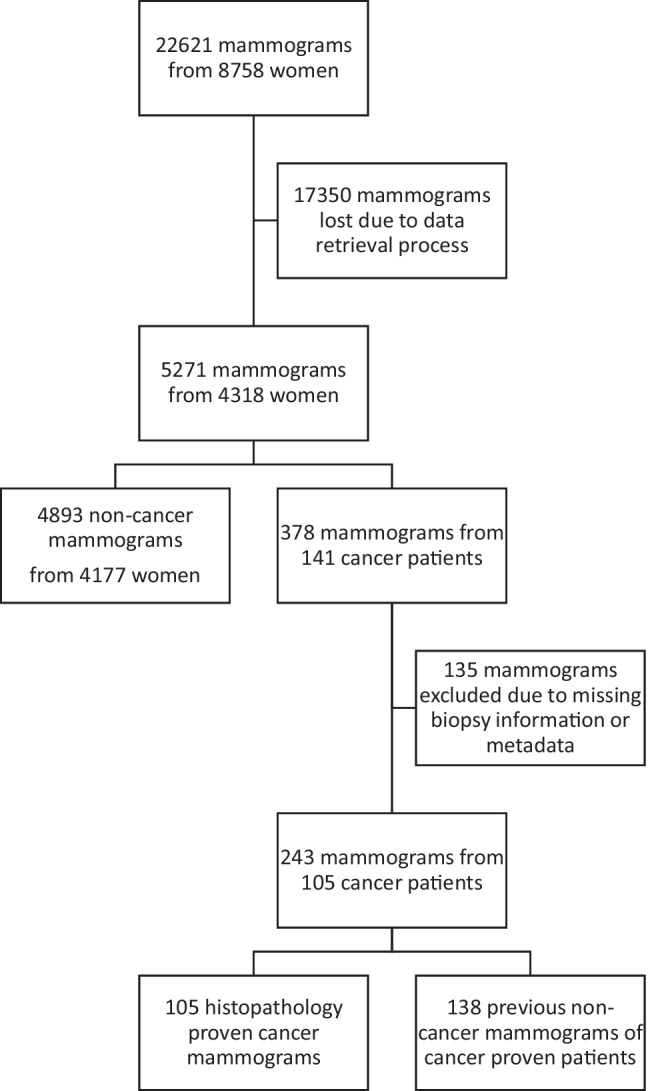
The flowchart of the used dataset and retrieval process

The images were sourced from the local PACS server of BMSP. However, the data had been archived within the PACS system since 2009, and the PACS system had not undergone updates until that time. Retrieving the data presented significant challenges owing to the antiquated version of the PACS system, resulting in the loss of numerous mammograms during retrieval. Despite seeking assistance from the PACS company’s professionals, the complete retrieval of all mammograms proved unattainable. To preserve patient confidentiality, all data underwent pseudonymization, with random identification numbers assigned during retrieval. A total of 22,621 mammograms were initially available, stemming from 8758 women. However, only 5271 mammograms from 4318 women were successfully retrieved due to the aforementioned technical limitations. Subsequently, 135 mammograms were excluded from the study due to missing information and suboptimal imaging quality. Consequently, the study encompassed 5136 mammograms from 4282 women, with 105 of these resulting in a diagnosis of breast cancer. A comprehensive elucidation of the dataset and retrieval process is provided in Fig. 1.

### Definitions

European Union Breast Cancer Screening Quality Guidelines were used in related definitions of BMSP [18]. (1) Interval cancer: Development of primary breast cancer in a woman within two years after a negative mammogram. (2) Missed cancer: False negative assessment, cancer diagnosis within first 30 days after a negative mammogram. (3) Screen-detected cancer: Cancer cases detected in the routine screening program.

### Artificial intelligence system

We used Lunit INSIGHT MMG version 1.1.7.1 (Lunit, Seoul, South Korea), a commercially available AI-based mammography interpretation software using convolutional neural network (CNN) algorithms. AI software evaluates MLO and CC views of each breast and creates a heatmap showing possible cancer lesions. AI software assesses a score for a lesion between 1 and 100 for each breast which reflects the likelihood of malignancy, and scores below 1 are given as low risk. The highest score between the breasts is defined as the risk score. The dataset used in the study has never been used in previously developed AI software.

### Evaluation with AI

**Fig. 2:**
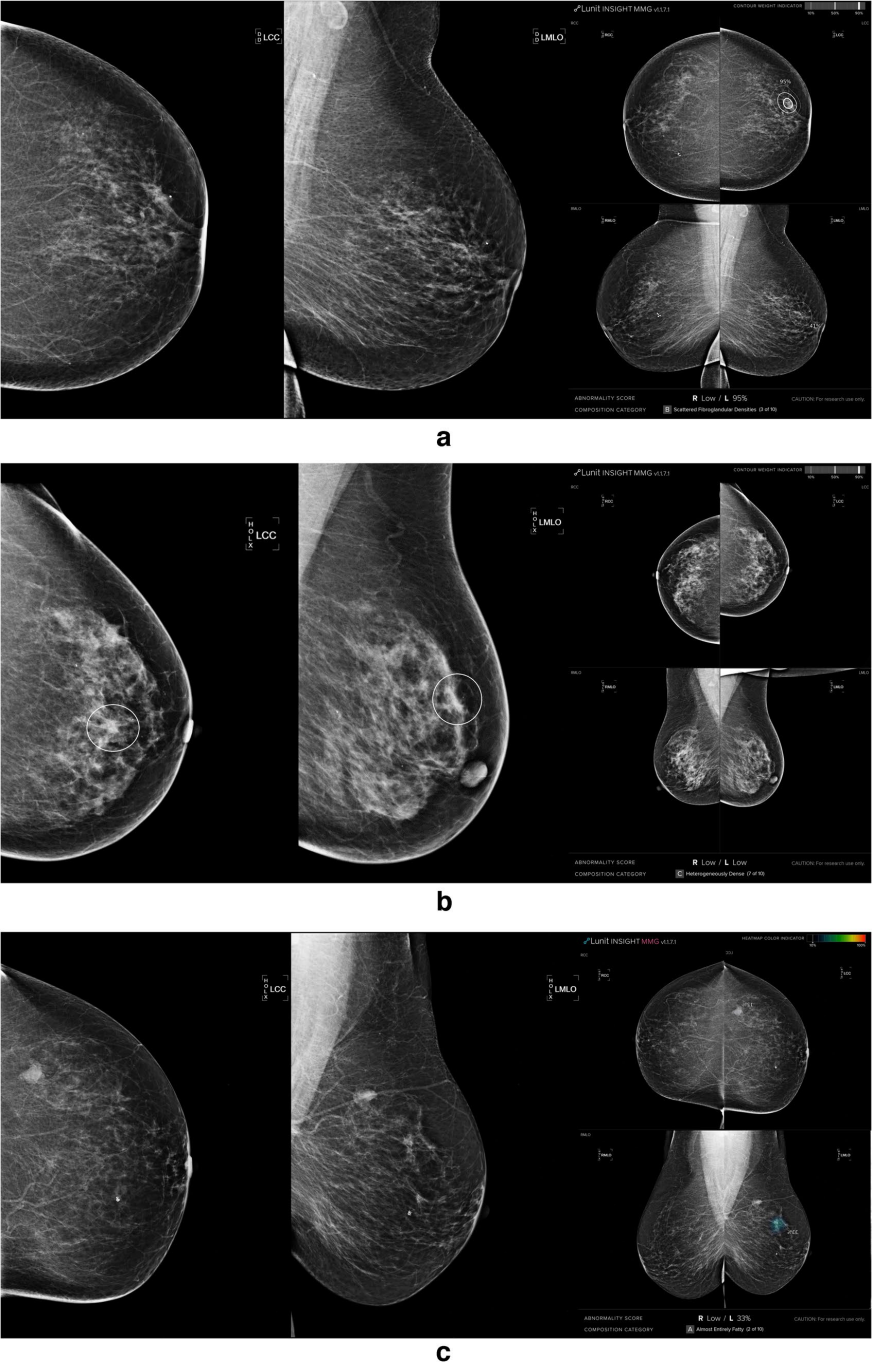
Examples of TP, FN, and FP mammograms. **a** A 62-year-old patient had a BI-RADS 5 lesion on the upper-outer quadrant of the left breast, with an AI system output showing a TP and successfully flagging the lesion. **b** A 59-year-old patient with BI-RADS 4 lesion on the 12 o’clock of the left breast (white circles), AI system did not flag any significant lesions showing FN. **c** A 58-year-old woman with no breast lesion with AI falsely flagged a lesion on the left breast as an example of an FP mammogram. TP = True Positive. FN = False Negative. FP = False Positive

**Fig. 3:**
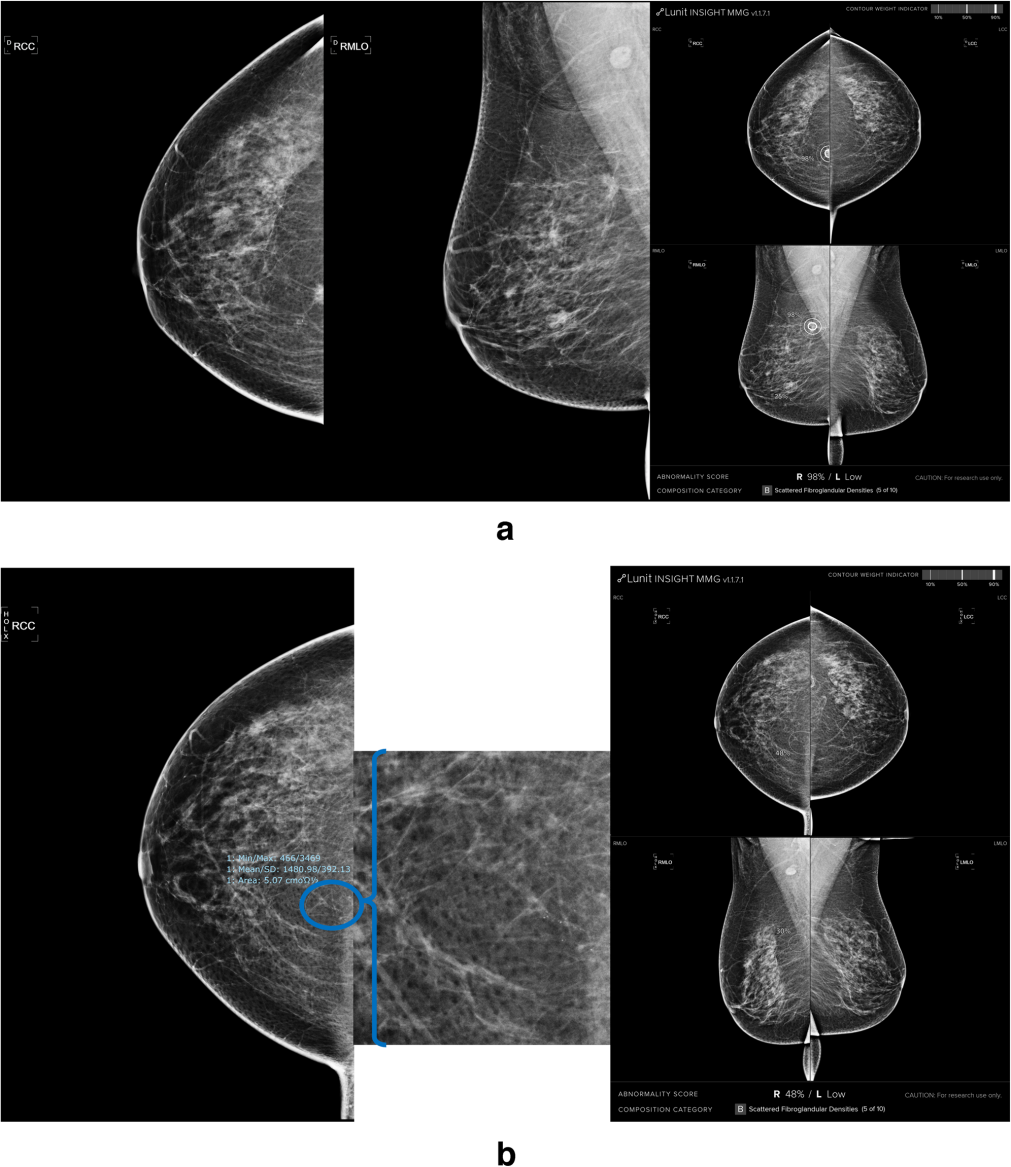
Example of longitudinal investigation. **a** The 52-year-old patient was diagnosed with a BI-RADS 4 lesion on the upper-inner quadrant of the right breast, with an AI system output showing a TP and successfully flagging the lesion. **b** No findings were found by radiologists in her mammograms from two years ago. AI system detected the same lesion on her mammograms from two years ago. Calcifications are better visualized with magnified view (blue circle and bracket)

All the retrieved mammography images were evaluated by AI software. Prior mammograms of cancer-detected mammograms were evaluated and noted for further longitudinal time analysis. An example of an AI system output is demonstrated in Fig. 2. True positive (TP), false negative (FN), and false positive (FP) examples are shown in Fig. 2. For the longitudinal evaluation of the cancer cases, we scrutinized the timing of cancer detection in prior mammograms, particularly in screening-detected patients. An example of longitudinal evaluation is given in Fig. 3. Missed, interval, and detected cancer cases were noted.

The risk assessments of the AI software were compared with the BI-RADS scores of the radiologists. BI-RADS scores were dichotomized: BI-RADS 1–2 as negative and BI-RADS 0–3–4–5 as positive as recommended by BI-RADS [17]. Ground truth was assessed with histopathology results.

A threshold was defined as given in the statistical analysis section by the “initial data set” of 5136 mammograms from 4282 women with 105 cancers. Mammograms were categorized based on their AI scores: those scoring below 1 were classified as negative, those scoring above or equal to the defined threshold were classified as positive, and those falling in between were categorized as flagged.

#### Workflow scenarios

For accurate simulation of used and proposed workflows, visits of patients with no cancer were bootstrapped to reach the actual cancer rate of BMSP 5.7 per 1000 [15], and a “simulated data set” was created. Bootstrapping was done with 10,000 iterations.

Three different workflow scenarios were simulated other than the original workflow of the BMSP, and the workload of radiologists was evaluated accordingly. In the actual workflow of BMSP, all mammograms were evaluated by two radiologists, with a third radiologist consulted in instances of discordance.

In scenario 1, a traditional workflow was simulated, often proposed by researchers, entailing an initial evaluation by a single radiologist complemented by AI software as the second reader [19].

In scenario 2, in addition to the scenario-1 approach, all flagged visits underwent an additional evaluation by a second radiologist.

In scenario 3, a triage algorithm with AI software was used to classify cases by green (mammograms with a risk score lower than and equal to 1), yellow (mammograms with a risk score between 1 and the threshold), and red (mammograms that had a risk score higher than or equal to the threshold). Green cases were labeled negative and were eliminated, whereas yellow and red cases underwent assessment by a single radiologist.

### Statistical analysis

All analyses were done with the R statistical language (Austria, R Core Team, version 4.1.0). A confidence level of 0.95 was considered significant. The normality of the data was assessed with the Kolmogorov–Smirnov test and skewness and kurtosis values. Variables fitting the normal distribution were described with mean and standard deviation; those not fitting were described with median and interquartile range (IQR). Categorical and ordinal variables were defined with absolute frequency. The receiver operating characteristics (ROC) curve and area under the curve (AUC) were analyzed. Youden’s Index was used to obtain a threshold for cancer identification. Differences in breast density, tumor size, mass and calcification types, cancer stage, molecular subtype, histological subtype and grade, nuclear grade, presence of necrosis, presence of lymphovascular invasion, multifocality, and type of surgery performed were analyzed with ANOVA, chi-squared test, and corresponding post hoc tests—workflows created for screening settings for workflow and accuracy comparison.

## Results

### Analysis of the initial data set

**Fig. 4:**
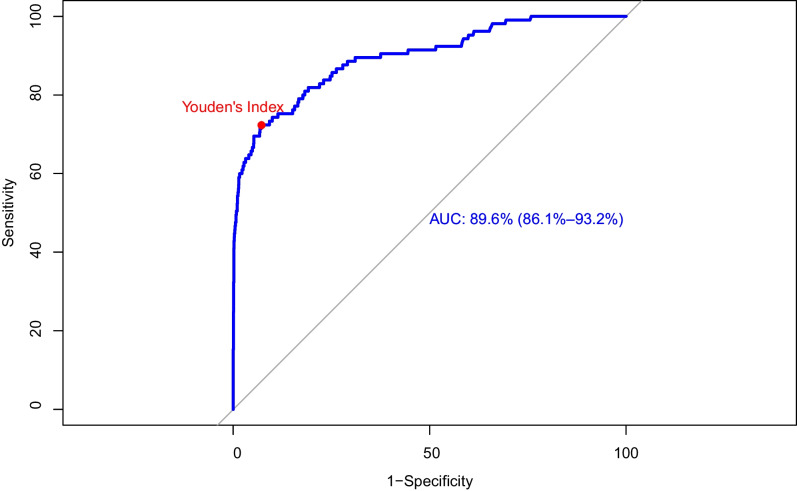
Receiver operating characteristic analysis and threshold were calculated using Youden’s index

The AUC of AI risk scores was 89.6% (86.1–93.2%, 95% confidence interval). Details of ROC are given in Fig. 4. Youden’s index of the ROC, yielding the highest sensitivity and specificity, was 0.65 at a score of 30.44, with 72.38% sensitivity and 92.86% specificity. Thus, the threshold was determined to be 30.44 and will hereafter be referred to as “the threshold”.

All the mammograms were labeled according to the threshold. Based on “the threshold,” 349 of the 4893 (7.13%) negative mammograms were false-positive. The mammograms of 29 of 105 (27.62%) cancer patients were false negatives.

**Fig. 5:**
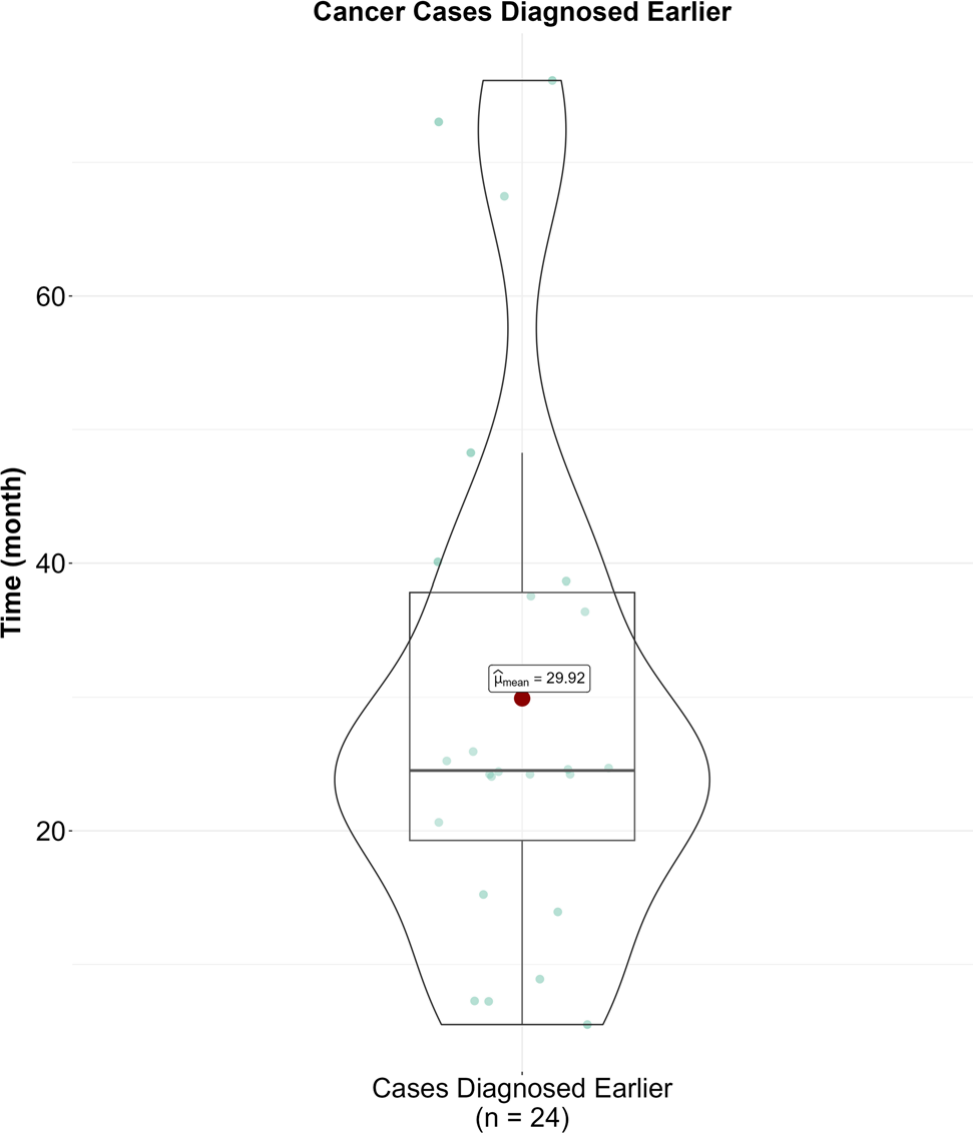
Boxplot demonstrating AI-led earlier diagnosis of prior mammograms of cancer patients

The prior 138 mammograms of these 105 cancer patients were further evaluated, and 35 prior mammograms of 24 cancer patients were labeled positive by AI. Three cancer cases were detected by AI 6 months earlier, which were labeled as BI-RADS 3 by BMSP. Furthermore, compared to BMSP detection, AI simulation resulted in the detection of three cancer cases one year earlier, ten cancer cases two years earlier, four cancer cases three years earlier, one cancer case four years earlier, and three cancer cases six years earlier. AI as a second reader could have led to earlier diagnosis with a mean ± standard deviation of 29.92 ± 9.67 months in 24 patients, as demonstrated in Fig. 5. Cancer and patient characteristics are further explained in Table 1. Distribution of the non-cancer, histopathology-proven cancer visits and characteristics of the cancer patients are given in Table 2.

**Table 1:** Characteristics of histopathology-proven cancers

Variables	Positive (*n* = 76)	Negative (*n* = 29)	*p* value
Density*	-	-	0.338
A	11 (91.67%)	1 (8.33%)	-
B	27 (75%)	9 (25%)	-
C	33 (67.35%)	16 (32.65%)	-
D	5 (96.25%)	3 (3.75%)	-
Tumor size**	13 (10.5)	15.25 (12.25)	0.702
Histological type*	-	-	0.56
Invasive Ductal Carcinoma	51 (72.86%)	19 (27.14%)	-
Invasive Lobular Carcinoma	8 (53.33%)	7 (46.67%)	-
DCIS	8 (80%)	2 (20%)	-
Intracystic Papillary Carcinoma	1 (100%)	0 (0%)	-
Invasive Cribriform Carcinoma	2 (100%)	0 (0%)	-
Microinvasive Carcinoma	1 (100%)	0 (0%)	-
Mucinous Carcinoma	1 (100%)	0 (0%)	-
Mixt Carcinoma	2 (100%)	0 (0%)	-
Neuroendocrine Differentiation	0 (0%)	1 (100%)	-
Tubular Carcinoma	2 (100%)	0 (0%)	-
Architectural distortion*	4 (5.26%)	1 (3.45%)	1
Focal asymmetry*	26 (34.21%)	6 (20.69%)	0.267
Mass*	-	-	-
Shape	-	-	0.977
Irregular	67 (72.04%)	26 (27.96%)	-
Round	6 (75%)	2 (25%)	-
Oval	3 (75%)	1 (25%)	-
Margin	-	-	0.425
Spiculated	40 (72.73%)	15 (27.27%)	-
Irregular	32 (69.57%)	14 (30.43%)	-
Well-defined	4 (100%)	0 (0%)	-
Microlobular	0 (0%)	0 (0%)	-
Obscured	0 (0%)	0 (0%)	-
Calcification (*n* = 84)*	68 (80.95%)	16 (19.05%)	-
Type	-	-	0.899
Pleomorphic	24 (77.42%)	7 (22.58%)	-
Heterogeneous	24 (82.76%)	5 (17.24%)	-
Amorphous	19 (82.61%)	4 (17.39%)	-
Fine linear	1 (100%)	0 (0%)	-
Distribution	-	-	0.845
Segmental	50 (81.97%)	11 (18.03%)	-
Regional	16 (76.19%)	5 (23.81%)	-
Diffuse	0 (0%)	0 (0%)	-
Clustered	1 (100%)	0 (0%)	-
Linear	1 (100%)	0 (0%)	-
Stage*	-	-	0.497
0	9 (81.82%)	2 (18.18%)	-
1	36 (72%)	14 (28%)	-
2a	15 (68.18%)	7 (31.82%)	-
2b	7 (100%)	0 (0%)	-
3a	7 (58.33%)	5 (41.67%)	-
3b	1 (100%)	0 (0%)	-
4	1 (50%)	1 (50%)	-
Surgery*	-	-	0.355
Mastectomy	16 (84.21%)	3 (15.79%)	-
Breast Conserving Surgery	60 (70.59%)	25 (29.41%)	-
Lymphovascular Invasion (yes)***	19 (25%)	10 (34.48%)	0.404
Necrosis (yes)***	8 (10.53%)	4 (13.79%)	0.852
Histological Grade*	-	-	0.282
1	15 (75%)	5 (25%)	-
2	42 (79.25%)	11 (20.75%)	-
3	19 (63.33%)	11 (34.67%)	-
Nuclear Grade*	-	-	0.282
1	15 (75%)	5 (25%)	-
2	42 (79.25%)	11 (20.75%)	-
3	19 (63.33%)	11 (34.67%)	-
Focality*	-	-	0.121
Multifocal	10 (100%)	0 (0%)	-
Multicentric	2 (67%)	1 (33%)	-
One Focus	64 (69.57%)	28 (30.43%)	-
Molecular Subtype*	-	-	0.19
Luminal A&B	57 (71.25%)	23 (28.75%)	-
HER-2 Positive	18 (81.82%)	4 (18.18%)	-
Triple Negative	1 (33%)	2 (67%)	-

*Presented with absolute frequencies between groups ** Presented with medians and interquartile ranges *** Presented with absolute frequencies within groups and totals

**Table 2:** Correlation of AI results with the radiologist evaluations

Variables	Negative (≤ 1%)	Flagged* (1% < x < 30.44%)	Positive (≥ 30.44%)
Non-cancer mammograms (*n* = 4893)	1928 (39.41%)	2616 (53.46%)	349 (7.13%)
Histopathology-proven cancer mammograms (*n* = 105)	5 (4.76%)	24 (22.86%)	76 (72.38%)
Detected by radiologists (*n* = 68)	1 (1.47%)	10 (14.71%)	57 (83.82%)
Interval (*n* = 29)	2 (6.9%)	12 (41.38%)	15 (51.72%)
Missed (*n* = 8)	2 (25%)	2 (25%)	4 (50%)

^*^Scores between 1 and threshold (30.44)

Of the 105 cancer cases in BMSP, 68 were screening-detected, 29 were interval cancers, and eight were missed. AI assigned a score equal to or higher than “the threshold” to 76 mammograms of 105 cancer patients (57 screening-detected (83.82%), 15 interval cancers (51.72%), four missed cancers (50%)), while 24 mammograms received a score lower than “the threshold” but were flagged for review. The remaining five mammograms scored less than or equal to 1 (Table 2).

#### Workflow scenarios with the “simulated data set”

**Fig. 6:**
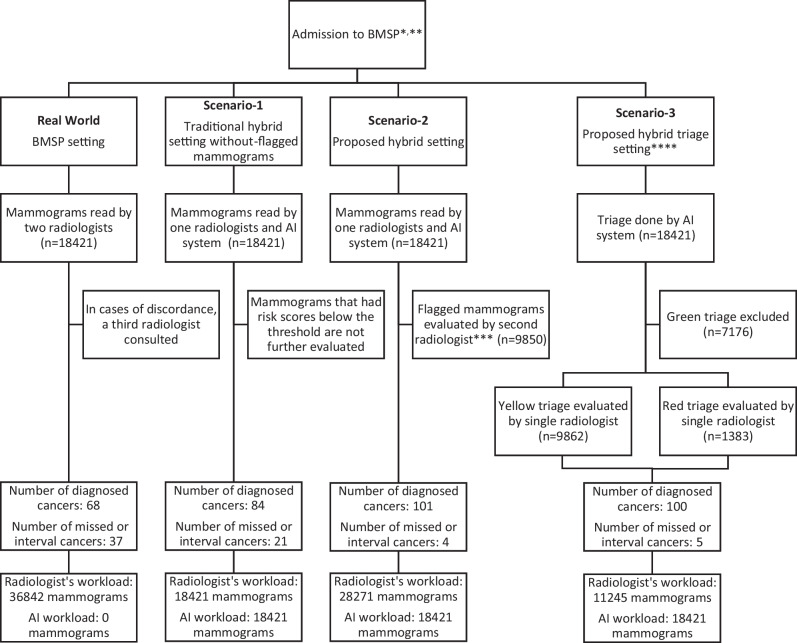
Simulations of workflows for BMSP

In total, the “simulated data set” consisted of 18,421 mammograms, which included 105 cases of breast cancer. In real BMSP setting two radiologists evaluated all 18,421 mammograms with a total workload of 36,842 evaluations. Of these mammograms, 68 of them were diagnosed with cancer, and 37 of them were missed or interval cancers. Proposed Scenario-1 reduced radiologist workload by half to 18,421, missed or interval cancers to 21, and increased diagnosed cancers to 84. Scenario 2 consists of a new hybrid setting, and it further increased diagnosed cancer cases to 101, with four missing or interval cancer cases. However, the workload of radiologists increased to 28,271 mammograms. The hybrid triage setting of Scenario-3 achieved similar results in terms of accuracy (30.5% increase from the BMSP setting) while decreasing workload (69.5% reduction from the BMSP setting) and detected 100 cancer cases with five missing or interval cancers. The workload of radiologists in scenario 3 was 11,245 mammograms. Detailed simulations of these workflows can be seen in Fig. 6. According to BI-RADS benchmarks, PPV1 should exceed 4.4%, and in scenario 3, findings indicate a PPV1 of 7.2%, surpassing the specified threshold. NPV for scenario 3 was 99.97% [17].

## Discussion

This study showed that AI can detect cancers with 72.38% sensitivity and 92.86% specificity when incorporated into a screening workflow as a triage mechanism. These findings are similar to the established benefits of using AI as a second reader in screening, which significantly increases sensitivity and specificity by incorporating the strengths of both AI and the radiologist [8, 11, 20, 21]. While AI could be an invaluable tool in distributing screening resources more effectively, this is in addition to its relatively improved performance in interval and missed cancers [8, 21]. We had previously shown that using AI increased detection rates of interval and missed cancers by 44.4% and 66.7%, respectively [8]. With the use of AI, sensitivity is greatly improved in breast cancer screening, particularly in cases where radiologists miss the cancer [7, 8, 22, 23]. This study also showed the impact of AI in early detection interval and missed cancers by 51.72% and 50%, respectively. This is in line with previous studies, which showed a detection rate of intervals of 20–50% [8, 9, 21]. Several clinical trials have been conducted to evaluate the performance of AI-based systems in breast cancer screening. Kim et al found that an AI-based computer-aided detection (CAD) system could detect breast cancers with a sensitivity of 89.7% and a specificity of 96.5% [24]. Hickman et al found that using an AI-based CAD system significantly increased invasive breast cancer detection, with a detection rate of 96.5% compared to 88.4% for radiologists alone [11].

AI-CAD systems commonly utilize risk scores or numeric values to assess mammograms, often in conjunction with the radiologist’s evaluation. However, it is important to acknowledge that different cohorts, demographics, imaging machines, and PACS systems may influence the risk thresholds employed in each study [25]. This emphasizes the need to develop region-specific AI models rather than relying on a universal approach. Establishing risk thresholds based on local criteria and implementing comprehensive training programs can enhance our understanding of breast cancers. Larsen et al examined three different screening scenarios, while our study explored four scenarios, yielding variable results with different thresholds [9]. Our scenarios revealed that AI would best function as a triage mechanism during breast cancer screening, leading to the best outcomes regarding early detection and workload reduction. While it is important to examine different screening scenarios, it is crucial to establish a workflow based on these scenarios to determine the optimal risk threshold for integrating AI into daily screening routines. Based on our experiences at BMSP, we propose the following outcomes: Firstly, AI-CAD systems can serve as a triage mechanism to distinguish the no-risk group from the at-risk group, reducing the workload of radiologists. At BMSP simulation, this approach achieved a triage rate of 38.9%. Implementing this strategy in clinical practice can facilitate broader screening readings and alleviate the demand for human resources. However, a legal framework should be developed for the triage system to address potential medicolegal issues. Secondly, cases falling between the no-risk group and the risk threshold should be classified as “flagged” cases, indicating a higher likelihood of false negatives (4.67%). These cases should receive a thorough evaluation by radiologists. Finally, patients receiving a risk score above the threshold should be considered true positive and referred for further diagnostic assessment. This approach enables the early identification of potential breast cancer cases, leading to improved treatment outcomes while reducing the workload.

At BMSP, this was equivalent to a workload reduction of 69.5% and an interval/missed cancer rate reduction of 30.5%. Numerous studies have investigated the impact of AI on workflow efficiency, consistently demonstrating similar outcomes in workload reduction with rates at an average of 20% with a maximum of 53% [26–29]. However, this approach involves a trade-off, as there is a risk of overlooking some cancers. In our study, the triage mechanism led to the misdiagnosis of five cancers. On the positive side, AI demonstrated successful detection rates of 93.1% and 75% for interval and missed cancers, respectively. The triage scenario ultimately identified 95% of all cancers observed during the 10-year screening period, surpassing the cancer detection rate in BMSP. A comparable scenario, as employed by Lang et al, revealed that using two different AI thresholds resulted in missing 7% or 1% of cancers, depending on the sensitivity level chosen [28]. They concluded that a slight decrease in sensitivity could enhance specificity. Considering the inherent occurrence of missed and interval cancers in screening programs, implementing AI triage presents an opportunity to alleviate human workload with an acceptable trade-off of missing a small number of cancers compared to real-world screening. This favorable trade-off supports the potential integration of AI triage into screening programs; however, the selection of thresholds emerges as a critical issue. Workload reduction is particularly important in countries with limited resources where the number of radiologists is often insufficient to provide broad coverage in a population-based screening.

A study by McKinney et al found that AI could reduce the workload of the second reader by 88% [25]. While this is quite a large amount, it only considers the second-reader. In our scenario 1, where AI serves as a second reader, we achieved a reduction in workload by 50%, whereas the triage scenario demonstrated 69.5%. In our triage approach, AI also functioned as a secondary reader for the remaining mammograms after triage. A recent meta-analysis indicates that employing AI as a second reader yields comparable outcomes, with a pooled AUC of 0.89 and readers at 0.85 [11]. In a recent prospective randomized study conducted by Lang et al, which compared double readers with a single reader and AI as a second reader, a 44.3% reduction in workload was observed with AI while maintaining comparable rates of cancer detection, recall, and false positivity [30]. Our approach represents a logical hybrid model, incorporating AI triage and AI as a second reader for the remaining mammograms post-triage.

Although AI has been widely studied regarding its detection capabilities, most studies evaluate screening data as a non-continuous, independent, and singular phenomenon. This could potentially lead to inaccurate results, especially with screening data, since cancer may not be radiologically evident as it develops over time [21]. This is evident in interval cancers, some of which may have been potentially present in preceding visits. To prevent false negatives of prior mammograms of cancer patients before they are diagnosed, a longitudinal analysis would be valuable for showcasing AI performance and risk scores over time. We have shown that using AI as a second reader in BMSP could have led to an earlier diagnosis in 24 patients by a mean of 29.92 ± 19.67 months. Considering the total number of cancers detected in BMSP, the early diagnosis of 22.9% of cancers in a screening program can potentially decrease morbidity and mortality [31]. Thus, the incorporation of AI as a triage tool reduces the workflow and dramatically enhances the early diagnosis rates, as much as up to a quarter of the detected cancers.

Similarly, Byng et al also had promising findings where subsequent cancers were detected in 25% of undiagnosed prior visits of interval cancers [21]. Despite the radiologist’s ability to detect cancer from previous visits, known as interval cancers, these findings frequently exhibit a bias due to the already established diagnosis of subsequent cancer. However, AI eliminates this bias if the exact cancers are not used for training, hence a more reliable result at the cost of increased false positives. Watanabe et al found that the use of AI-CAD software significantly improved radiologists’ cancer detection rate, with an increase ranging from 6 to 64% (mean 27%) and a negligible increase in false-positive recalls [22]. At BMSP, the ratio of false positives we acquired in the flagged and above-threshold groups is 7.13%. This may slightly increase the demand for further diagnostic studies. Still, with the ability to analyze mammograms more quickly and accurately, AI-based systems would be able to detect breast cancers at an earlier stage when they are more treatable.

## Limitations

This study was conducted retrospectively, and screening scenarios should be validated with prospective studies of similar constructs. Secondly, due to unforeseen issues during data migration, we were unable to include all the mammography images of patients screened at BMSP in our analysis. To overcome this, we bootstrapped the healthy visits from patients not diagnosed with cancer for at least two subsequent rounds of screening.

## Conclusion

In conclusion, the AI system demonstrated high sensitivity and specificity in cancer detection in screening mammograms. AI contributes to the early detection of interval and missed cancers, reducing human errors. Furthermore, the potential of AI in identifying up to 23% of cancers earlier in prior mammograms holds promise. This study also underscores the significance of a well-considered strategy for integrating AI into screening programs. Adopting AI as a triage mechanism within screening workflows could effectively reduce workload close to 70% and augment the timely detection of cancer.

## Abbreviations

AI: Artificial intelligence
AUC: Area under the curve
BI-RADS: Breast Imaging-Reporting and Data System of the American College of Radiology
BMSP: Bahcesehir Mammographic Screening Program
CC: Craniocaudal
CNN: Convolutional neural network
IQR: Interquartile range
MLO: Mediolateral oblique
ROC: Receiver operating characteristics

## Declarations

### Guarantor

The scientific guarantor of this publication is Erkin Aribal.

### Statistics and biometry

One of the authors has significant statistical expertise.

### Methodology

• retrospective

• observational

• performed at one institution
